# Exercise During Adolescence Attenuated Depressive-Like Behaviors and Hippocampal Mitochondrial Dysfunction Following Early Life Stress in Adult Male Rats

**Published:** 2018

**Authors:** Forouzan Fattahi Masrour, Maghsoud Peeri, Mir Jamal Hosseini, Mohammad Ali Azarbayjani

**Affiliations:** a *Department of Exercise Physiology, Central Tehran Branch, Islamic Azad University, Tehran, Iran. *; b *Zanjan Applied Pharmacology Research Center, Zanjan University of Medical sciences, Zanjan, Iran.*; c *Department of Pharmacology and Toxicology, Faculty of Pharmacy, Zanjan University of Medical Sciences, Zanjan, Iran.*

**Keywords:** Adolescence, Depression, Early life stress, Fluoxetine, Mitochondria, Voluntary running wheel exercise

## Abstract

In this study, we assumed that treating animals with an antidepressant agents or voluntary running wheel exercise (RW) during adolescence may have beneficial outcomes against early life stress (ELS) which could be effective on behavior and mitochondrial function. Evidence indicated that ELS has deleterious impacts on brain and increases the risk of brain disorders such as depression. Maternal separation stress (MS) model to male rats (postnatal day or PND2-PND14) were performed to determination of depressive-like behaviors using the forced swimming test, splash test, and mitochondrial function in the hippocampus. Treating MS rats with both RW and fluoxetine (5 mg/kg/day, i.p) during adolescence (PND30-PND60) produced antidepressant-like effects in animals and attenuated the negative effects of ELS on hippocampal mitochondrial function in adult male rats. The results of the present study showed that (none) pharmacological treatments during adolescence are able to produce therapeutic effects against adverse effects of ELS on behavior and mitochondrial performance. These results showed the importance of adolescence as an important period of life and the long-lasting effects of ELS on hippocampal mitochondrial function which can suggest the possible contribution of abnormal mitochondrial function in pathogenesis of depression following experiencing ELS.

## Introduction

Experiencing stress during childhood is associated with the development of psychiatric disorders such as depression. Depression is a serious debilitating mental disorder accompanying with high morbidity and mortality among sufferings (1, 2). Recent evidence indicates that stress-induced mitochondrial dysfunction contributes to the pathogenesis of depression (3-5). Mitochondria is the main source of energy production in brain cells and its abnormal performance leads to the massive formation of reactive oxygen species (ROS), initiation of immune-inflammatory responses, and consequently, impairment of energy hemostasis in the brain (5-7). Preclinical studies using animal models of depression (such as social isolation stress and chronic mild stress) have shown that stress-induced behavioral abnormalities are associated with mitochondrial dysfunction. So, antidepressant therapies could effectively attenuate the negative effects of stress on mitochondrial function in the brain (8-10). Since timing and duration of stress exposure are determinant factors contributing to negative effects of stress on brain, it should be considered that mitochondria during early stages of life are not mature and functional as they are in adulthood stage (11, 12). Developing brain undergoes significant growth and maturation in early stages of life and also, mitochondria provides required energy for these developmental processes. Recent evidence indicates that developing brain is susceptible to stressful events due to its high content of poly unsaturated fatty acid (PUFA), higher iron levels, higher activity of mitochondrial respiratory activity, and immature mitochondria pool (13, 14). Based on this evidence, we hypothesized that behavioral abnormalities following experiencing early life stress may be associated with abnormal mitochondrial function in the regions of the brain relevant to depression such as hippocampus (HIPP). 

Maternal separation stress (MS) is a standard model system for investigating the effects of stress during early stages of life on brain and behavior (15, 16). We recently showed that voluntary running wheel exercise during adolescence not only could effectively reverse the depressive-like behaviors in adult rats exposed to MS, but also succeeded to attenuate the immune-inflammatory responses in the HIPP of subjects (17). It has been well evident that regular physical activity and exercise have significant positive impact on different aspects of mental health such as mood, cognition, self-esteem, and quality of life (18, 19). Exercise exerts its anti-stress effects through a variety of mechanisms such as promoting the mitochondrial function and enhancing the antioxidant capacity in the brain (20, 21). In the present study, we applied MS to male rats to investigate whether 1) experiencing MS is able to alter behavior and mitochondrial function in the hippocampus and prefrontal cortex of adult rats, and 2) voluntary running wheel exercise (RW) during adolescence mitigates the adverse effects of MS on behavior and mitochondrial function in the HIPP.

## Experimental

*Animals and housing conditions:*

Pregnant Albino Wistar rats (Pasteur institute, Tehran, Iran) were purchased and kept under standard laboratory conditions with free access to food and water at animal facility center of the Department of Exercise Physiology, Central Tehran Branch, Islamic Azad University. Neonatal maternal separation protocol was applied to animals based on previous studies (17, 22). In brief, postnatal day 0 (PND 0) was considered as the day of birth and male pups were subjected to MS stress. The pups were briefly handled and then were separated from their mothers for 3 h every day during PND 2 - PND 14, beginning at 09:00 a.m. At the PND 21, male animals were housed in groups (four rats / cage) until the (PND 28). Institutional guidelines were strictly followed for animal care and use (Department of Exercise Physiology, Central Tehran Branch, Islamic Azad University, Tehran, Iran).

*Materials and treatments:*

Fluoxetine hydrochloride (Sigma USA) was dissolved in saline and solution was prepared instantly before the injections. Fluoxetine (FLX) was administered intraperitoneally (i.p.) in a constant volume of 5 ml/kg body weight to both MS and the control rats from PND 28 to PND 60. We used chronic administration of FLX (5 mg/kg) to MS and control rats because it has been reported as an effective treatment for modifying depressive-like behaviors in rodents (17, 23).

*Running wheel (RW) exercise:*

At the PND 21, the animals were randomly subjected to exercise protocols including RW exercise. In case of RW exercise, the MS and control rats were allowed to adapt to the RW apparatus for 1 week. The exercise protocol was carried out based on the method previously described (17, 24). In order to avoid the effects of social isolation, two rats in a cage were kept apart with a perforated Plexiglas separator between them that facilitated communication between the rats. The control group of MS rats were similarly handled but had no access to RW. Each activity wheel was made of Plexiglas (circumference = 105 cm, width = 10 cm, Novidan Tab, Iran) which was freely rotated against a resistance of 5g. Each wheel was connected to a magnetic switch that was linked to a counter placed outside of the animals’ cage and monitored the revolutions per hour. The rats had free access to the activity wheel 24 h a day for 32 days until PND 60, and daily running distance (km) was recorded.

*Behavioral tests*

*Open-field test (OFT)*

This test was used to evaluate the effects of housing and treatment conditions on number of squares crossed (horizontal activity) and the number of rearing (vertical activity) based on our previous studies (8, 17). 

*Forced swimming test (FST)*

The passive behavior (immobility time) was defined as the main behavioral marker in depression according to Porsolt *et al*., 1978 (25) in cylindrical glass tank (50×20 cm in diameter) containing 30 cm of water (23 ± 1 °C). Two swimming sessions were performed with an initial 15 min ‘pre-test’ followed by a 5 min ‘test’ after 24 h. 

*Splash test*

The anhedonic state and self-care difficulties in animals in splash test as described by our previous studies based on recording the first grooming latency and the time spent grooming for a period of 5 min (8, 17). A 10% sucrose solution was squirted on the dorsal coat of animals in their home cage. The first grooming latency and the time spent grooming were recorded for a period of 5 min.

*Mitochondrial functional tests*

*Mitochondrial preparation*

Different sets of animals from those used in the behavioral experiments were used to investigate the mitochondrial function and oxidative state in the HIPP. Hippocampi were quickly dissected out on ice after decapitation, soaked in the liquid nitrogen, and kept at -80 °C until the assays. Measurement of the protein concentration was performed based on Coomassie blue protein-binding method using bovine serum albumin (BSA) as the standard. The extraction of mitochondrial fractions was carried out as previously described (26, 27). In order to confirm the purity of mitochondria, succinate dehydrogenase (SDH) activity was measured (28). 

*Adenosine triphosphate (ATP) assay*

The ATP levels were measured using luciferase enzyme as described in our previous work (29, 30). Bioluminescence intensity was measured using Sirius tube luminometer (Berthold Detection System, Germany).

*Measurement of reactive oxygen spices (ROS) formation*

In order to measure the mitochondrial ROS formation, isolated HIPP mitochondria was incubated with DCFH-DA(10 µM) in respiratory buffer using the BD Biosciences FACS Caliber TM flowcytometer using a 530-nm band pass filter (FL-1 channel) (31, 32).

*Reduced glutathione (GSH) level assay:*

Glutathione levels were determined using of 5, 5′-dithiobis- (2-nitrobenzoic acid) or 5′-dithiobis-2-nitrobenzoic acid (DTNB)as a reagent with developed yellow color at 412 nm using a spectrophotometer (UV-1601 PC, Shimadzu, Japan) expressed as µM/mg protein based on the calibration standard curve (32, 33).

*Experimental design:*

In this study, the animals were divided into six experimental groups each contains 6-8 male rats as follows; 1) Control group, 2) MS group, 3) Control group + RW treatment, 4) Control group + FLX treatment 5) MS+FLX treatment, and, 6) MS+RW treatment. After the termination of exercise protocol and FLX treatment at PND 60, the animals were subjected to behavioral tests. Also, at PND 60, the animals in the experimental groups were sacrificed under mild anesthesia and HIPP tissues were freshly collected for mitochondrial activity assays as described above. 

*Statistics*

Comparison between the groups was analyzed using *t*-test and one-way ANOVA followed by multiple comparison tests in the Graph-pad prism software (version 6). P ˂ 0.05 was considered statistically significant.

## Results

In this work, we decided to investigate the impacts of chronic RW exercise on depressive-like behaviors induced by MS models in adult male rats. The one-way ANOVA analysis yielded a significant effect of manipulations on animals’ behavior between groups. in the FST [F (5, 36) = 33.946, P < 0.001] and splash test [F (5, 36) = 13.215, P < 0.001]. Table.1 shows that MS rat group has increased immobility time in comparison with control groups (^***^P < 0.001). Also, MS+ RW and MS+FLX groups showed decreased immobility time compared MS control group in Tukey’s post analyses (^###^P < 0.001). It should be noted that there was no difference between the RW-control and FLX- control rats in the immobility time (P > 0.05) compared to control counterparts. The effects of MS on the grooming activity time in the splash test showed a significant decrease in duration of grooming activity compared to control group (^***^P < 0.001). Our data presented on Table.1 reveals that there is no significant difference in the number of squares crossed (Horizontal activity) between all groups (P > 0.05) indicating no change in the locomotion of animals in the OFT.

**Table 1:** Effect of RW exercise and FLX on depressive-like behaviors induced by MS in adult rats: Effect of chronic adolescent RW exercise as well as chronic FLX treatment, on the immobility time in the FST, grooming activity time in the splash test and number of squares crossed in the OFT were determined in different animal groups. Values are expressed as the mean ± SD and were analyzed using one-way ANOVA followed by tukey’s post-test analysis (n = 6-8 in all groups).

**Groups**	**FST**	**Splash test**	**OFT**
	**Immobility time (Sec)**	**Grooming time (Sec)**	**Horizontal activity ( number of crosses)**
Control	80±19	123±27	110±11
RW	76±12	104±23	98±12
FLX	83±17	96±20	104±32
MS	174±12***	23±8***	79±32
MS+RW	74±14###	83±39###	93±24
MS+FLX	101±21###	91±25###	100±13

* *P *< 0.05,

** *P *< 0.01 and

*** *P *< 0.001 compared with the control group;

# *P *< 0.05,

## *P *< 0.01, and

### *P *< 0.001 compared with the MS groups.

**Figure 1:**
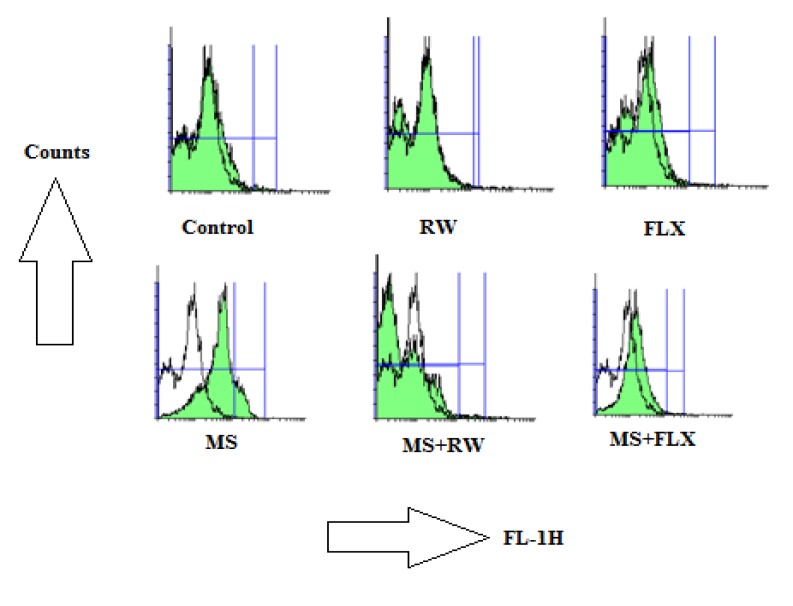
Effects of MS on ROS formation in the hippocampus: The ROS in each sample was read with 485 nm excitation and 520 nm emission using a fluorimeter after 20 min in different animal groups. The signs for increased ROS formation in flowcytograms are shifting the ROS peak to the right and increasing of AUC

**Figure 2:**
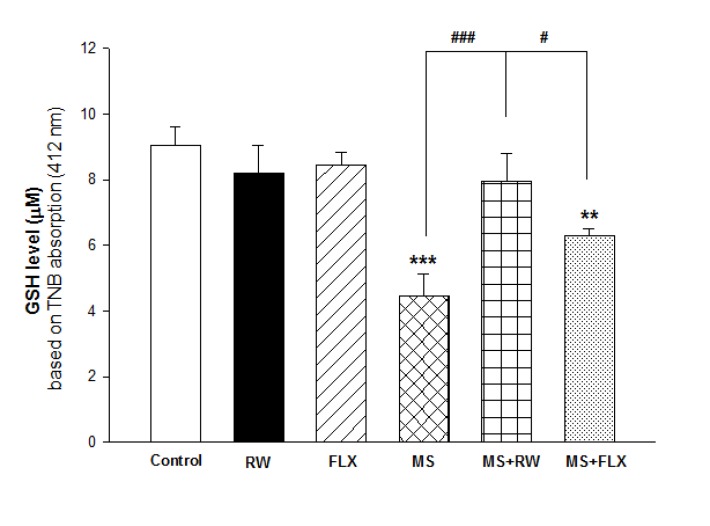
Effect of MS on GSH level in the hippocampus: Values are expressed as Mean ± S.D. and were analyzed using one-way ANOVA followed by tukey’s post hoc tests **P < 0.01; ***P < 0.001 compared with control group; # *P *< 0.05; ### *P *< 0.001 compared with MS group

**Figure 3:**
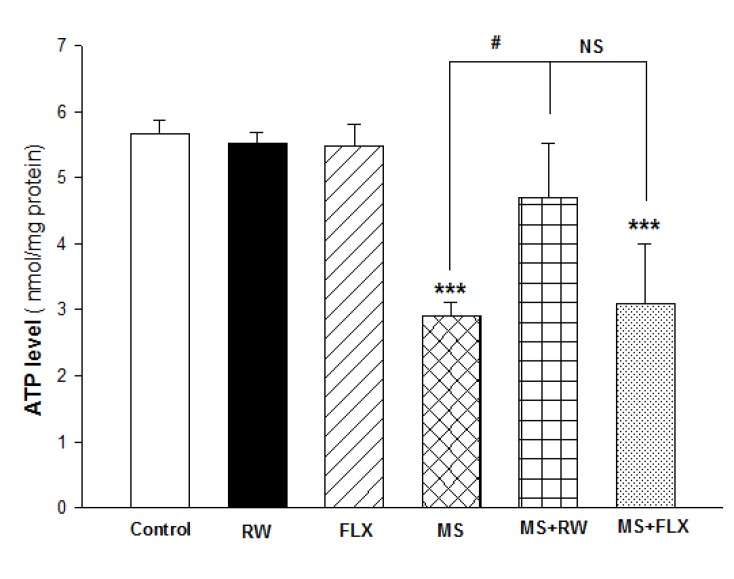
Effect of MS on ATP level in the hippocampus: Values are expressed as Mean ± S.D. and were analyzed using one-way ANOVA followed by tukey’s post hoc tests *** *P *< 0.001 compared with control group; # *P *< 0.05compared with MS group

In biochemical assay, as shown in Figure 1, MS rats showed higher rate of ROS formation in the HIPP of MS group after 20 min incubation. Increasing of mitochondrial ROS formation in flowcytograms is shown as rightward shifting of the ROS peak. As shown in Figure 1, there is no increase in ROS production in different rat groups compared to control groups obtained from HIPP mitochondria (P > 0.05) (Figure 1) suggesting the efficacy of FLX and RW treatment on mitochondrial ROS production. Also, it indicates that there is no difference between the control groups treated with FLX or RW.

As shown in Figure 2, one-way ANOVA analysis showed that mitochondrial GSH levels in MS and MS+FLX rat group were deceased compare to the control rat group [F(5,12) = 22.198; *** P < 0.001 and** P <0.01, respectively) suggesting the role of oxidative stress in MS treated groups. Also, FLX and RW treated MS rats presented increasing of GSH level (antioxidant power) when compared to MS rats (#P < 0.05 and ###P<0.001, respectively). As shown in Figure 2, the effect of RW+MS and FLX+MS rat groups on GSH levels were increased to 7.96 ± 0.83 and 6.29±0.23 µM/mg protein compared to the MS rats (4.46 ± 0.67 µM/mg protein), respectively. It seems that the effect of RW on GSH level was higher than FLX in MS rat groups.

The effects of MS on hippocampal ATP mitochondrial levels are demonstrated in Figure 3. One-way ANOVA analysis showed a significant difference in ATP level between MS and MS+FLX compared to the control [F (5,12) = 16.748; *** P < 0.001]. These results showed chronic RW exercise has a positive significant effect on ATP level in isolated mitochondria obtained from HIPP compared to MS rats (#P < 0.05). However, there was no significant difference in the ATP level between MS and MS+FLX rats (P > 0.05).

## Discussion

Our results showed that experiencing MS during early stages of life induce depressive-like behaviors in adult male rats. Further, we found that administration of fluoxetine (a common prescribed antidepressant) or voluntary RW activity during adolescence could attenuate the negative impacts of MS on animal behaviors. Furthermore, it revealed that MS is able to induce mitochondrial dysfunction in the HIPP of adult male rats. Moreover, treating of stressed rats with fluoxetine/voluntary RW activity in adolescence improves mitochondrial function in the HIPP.

There are a lot of evidences reporting that experiencing chronic stress in all stages of life is considered as a potent risk factor for the development of mental disorders such as depression (2, 11). Focusing on the etiology of depression, recent evidence indicates that mitochondrial bioenergetics dysfunction contributes to the pathogenesis of depression through several pathways such as production of oxidative stress, initiation of inflammatory pathways, and altering the neuroplasticity (3-5, 7). In this work, we showed that MS induced abnormality in mitochondrial activity in the HIPP of the male rats in adulthood. The rate of ROS formation results showed that in the hippocampi mitochondria, the MS rats produced higher levels of ROS in comparison with the control groups indicating that MS during early stages of life is able to induce overproduction of ROS by hippocampal mitochondria. Overproduction of ROS is associated with the abnormal performance of mitochondria and oxidative stress in the brain cells (34). In line with these results, we showed that experiencing MS results in a significant decrease in the level of GSH in hippocampal mitochondria confirming that increased ROS production negatively affects the GSH as the most important antioxidant system in the CNS. Previous studies have shown that patients with psychiatric disorders have reduced levels of GSH in their brains (35). In addition, ATP measurement in the brain tissues showed that MS decreased the level of ATP in the HIPP. This finding suggests that MS is able to disrupt the energy metabolism in the HIPP of adult rats. Results of our lab and others have previously shown that subjecting rodents to stressful events (such as social isolation stress and chronic mild stress) is able to induce mitochondrial dysfunction in the brain (8, 10, 30). In addition, recent investigations demonstrated that early-life stress has detrimental effects on mitochondrial function in the brain (36, 37).

It has been well-evident that stress-induced oxidative stress plays an important role in the development of mental disorders. Indeed, oxidative stress is the result of imbalance between antioxidant system and ROS production. Under chronic stressful conditions, abnormal mitochondrial function is able to produce high amounts of ROS that (in) directly alters neurotransmission system (such as glutamate system), Hypothalamic-pituitary-adrenal (HPA) axis deregulation, and initiates apoptotic processes and inflammatory responses (34, 38, 39). All these mechanisms contribute to the pathophysiology of depression and our results are in agreement with previous investigations which highlighted the long-lasting impact of Early life stress (ELS) on brain mitochondrial function and behavior (40-42). Further. In our study MS targeted the brain at early stages of life when the brain is still developing and mitochondrial function and antioxidant system in the brain is not as mature as adults. Thus, experiencing stress during this period may have more detrimental effects on mitochondria and brain (12, 13). However, in this study, we assumed that adolescence may be an appropriate period in which the brain is still developing and treating MS animals with (none) pharmacological therapies (chronic FLX and RW exercise) may attenuate the effects of MS on adulthood. Our results showed that treating animal with both chronic FLX and voluntary RW during adolescence effectively mitigated the adverse outcomes of ELS on hippocampal mitochondrial function. This treatment improved antioxidant system (increased GSH levels), reducing the excessive ROS production, and increased ATP levels (energy metabolism, except for FLX) in the HIPP. There are few studies which target the adolescence as an appropriate time to treat stressed animals in order to investigate the effects. A study by Della and colleagues recently done has shown that MS induced mitochondrial dysfunction in the brain and treating animals with tianeptine in adulthood had therapeutic effects and decreased the mitochondrial dysfunction (36). Another study using respiratory control index (RCI), a method for mitochondrial activity evaluation, showed that MS stress had no effect on mitochondria in prefrontal cortex but striatum was affected (37). However, a study by Daniels *et al*. showed the protective effects of exercise during adolescence on protein expression in the brain (43). We also previously showed that voluntary (but not obligatory treadmill) RW exercise reversed the depressive like behaviors in adult MS rats, and these effects were accompanied by the downregulation of genes associated with innate immunity such as toll-like receptor 4 and 2 in the HIPP (17). It is important to note that either FLX or RW did not alter the mitochondrial function in the HIPP of adult rats. Our laboratory and others have recently shown that treating animals with chronic FLX during adolescence has no effect on mitochondrial function in adulthood (8, 44).

We also investigated the depressive-like behaviors of animals using validated behavioral tests such as FST and splash test. We found that MS increased the immobility time in the FST without altering the locomotion of animals in the OFT. These results suggest that MS provokes behaviors associated with behavioral despair in rats (45). Splash test is behavioral test that was designed to evaluate the self- care and anhedonia in rodents, respectively (46-48). Our results showed that stressed animals exhibited decreased grooming activity time in the splash test suggesting the ability of MS to induce depressive-like behaviors in animals. However, treating stressed rats with chronic FLX and RW in adolescence efficiently attenuated the depressant effects of ELS on adult rats. These treatments decreased the immobility time in the FST and increased the grooming activity time in the splash test without altering the locomotion of the animals in the OFT. Previous results from other laboratories are in line with our results that treating animals with FLX (or other antidepressants) and exercise are able to alleviate the depressive-like behaviors of the animals exposed to ELS (49, 50). It is important to note that we did not have enough female pups to do the same experiments on them and unfortunately we did not succeed to show the effects of adolescent voluntary RW exercise/ chronic FLX on the negative effects of MS on depressive-like behaviors in adulthood. Also, we should note that MS has been shown to have several behavioral and cognitive effects on rodents such as alteration in memory and learning, seizure activity, anxiety and fear, pain response and others that evaluating all of these behaviors was not possible for us and we only focused on depressive-like behaviors, albeit we observed anxiogenic effects of MS in OFT (data not shown) (51-54).

In conclusion, our results demonstrated that MS, as a standard animal model of ELS, induces depressive-like behaviors and abnormal hippocampal mitochondrial function in adult male rats. These negative effects of MS were attenuated by adolescent voluntary RW exercise or chronic FLX treatment. Our results highlight the importance of adolescence as a stage of life in which treating animals by pharmacologic agents or environmental manipulations during this period has promising positive effects against ELS outcomes on adulthood.
